# Single-Round Patterned DNA Library Microarray Aptamer Lead Identification

**DOI:** 10.1155/2015/137489

**Published:** 2015-05-14

**Authors:** Jennifer A. Martin, Peter A. Mirau, Yaroslav Chushak, Jorge L. Chávez, Rajesh R. Naik, Joshua A. Hagen, Nancy Kelley-Loughnane

**Affiliations:** ^1^Human Effectiveness Directorate, 711 Human Performance Wing, Air Force Research Laboratory, Wright-Patterson Air Force Base, Dayton, OH 45433, USA; ^2^The Henry M. Jackson Foundation for the Advancement of Military Medicine, 6720A Rockledge Drive, Bethesda, MD 20817, USA; ^3^Materials and Manufacturing Directorate, Air Force Research Laboratory, Wright-Patterson Air Force Base, OH 45433, USA; ^4^UES Inc., 4401 Dayton-Xenia Road, Dayton, Dayton, OH 45433, USA

## Abstract

A method for identifying an aptamer in a single round was developed using custom DNA microarrays containing computationally derived patterned libraries incorporating no information on the sequences of previously reported thrombin binding aptamers. The DNA library was specifically designed to increase the probability of binding by enhancing structural complexity in a sequence-space confined environment, much like generating lead compounds in a combinatorial drug screening library. The sequence demonstrating the highest fluorescence intensity upon target addition was confirmed to bind the target molecule thrombin with specificity by surface plasmon resonance, and a novel imino proton NMR/2D NOESY combination was used to screen the structure for G-quartet formation. We propose that the lack of G-quartet structure in microarray-derived aptamers may highlight differences in binding mechanisms between surface-immobilized and solution based strategies. This proof-of-principle study highlights the use of a computational driven methodology to create a DNA library rather than a SELEX based approach. This work is beneficial to the biosensor field where aptamers selected by solution based evolution have proven challenging to retain binding function when immobilized on a surface.

## 1. Introduction

Aptamers are oligonucleotide molecular recognition elements selected through a synthetic iterative evolutionary process termed SELEX (Systematic Evolution of Ligands by EXponential enrichment) [1, 2]. Since their discovery, aptamers have been selected for a variety of targets from ions to whole cells and implemented in applications such as therapeutics, purification, or as biosensor detection ligands. The use of aptamers for these applications over other types of recognition elements is warranted by their well-reported advantages, including the ease and reproducibility of chemical synthesis, simplicity of modifying aptamers with fluorescent tags or surface immobilization chemistries (amine, biotin, etc.), and relatively high stability to degradation [3].

Despite the advantages of aptamers, several significant drawbacks are inherent to the standard SELEX process. One example is the timescale of aptamer selection, which typically requires an average of 12 cycles and a minimum of 2–6 months, not including initial optimization processes, validation of aptamer candidates, or structural analysis [4, 5]. This is typically a result of the low partitioning efficiency (the ability to separate binding sequences from nonbinders in a selection round) of conventional partitioning methods used in SELEX [6]. Furthermore, SELEX suffers from polymerase chain reaction (PCR) bias, where the PCR has been reported to amplify oligonucleotides unequally, resulting in an inaccuracy of comparative representation within a pool as the selection progresses [7]. A second form of bias is introduced by the cloning and Sanger sequencing method used for aptamer identification. The sequences reported likely reflect the most abundant sequences present but may not report those that have artificially lower numbers due to factors such as PCR bias or cloning efficiency or due to low sampling of the entire sequence space (diversity) of the final pool [8].

Several groups have proposed using DNA microarrays to address the possible SELEX biases and expedite the aptamer identification process [9–11]. DNA microarrays function by identifying locations of fluorescently labeled targets and correlating them with the position of known sequences covalently synthesized on the array. The arrays are fully customizable so the user can define the exact sequences of interest produced on the arrays. A single microarray experiment can be completed in less than one day, with an additional 1-2 days for data analysis. This characteristic is a function of the improved partitioning efficiency available by covalently linking the sequences to the surface. Higher stringency conditions can be applied to identify sequences with more ideal binding properties. PCR, cloning, and sequencing are not required since known sequences are in predefined locations. Also, microarrays are particularly useful for identifying aptamers for biosensor applications, since the response of an aptamer selected by solution-based SELEX may be significantly diminished when it is tethered to a sensor surface [12–14].

A major drawback of microarray use is that the highest density arrays have a maximum of ~10^6^ sequences, in contrast to SELEX methods, which evolve from an initial library of ~10^15^ sequences. However, combinatorial drug-screening libraries successfully identify binders with only 10^3^–10^6^ different compounds in the starting library due to the diversity of the functional groups of the compounds [15]. Extending this premise to oligonucleotides, it has been determined that the probability for a sequence to bind a target improves with increasing structural complexity [16]. This means unstructured sequences or oligonucleotides that form simple structures, such as those in a random oligonucleotide library, have reduced potential to show any type of function. Constituents of the random pool consist of mostly unpaired regions combined with short (low stability) stem-loop structures, and the probability of containing an abundance of more complex, high-affinity aptamers in the starting random library is low [17]. Several groups have explored this issue experimentally by optimizing the SELEX starting library to contain more complex, partially structured sequences [18–20]. These studies showed that the partially structured libraries provided more sequences that bind the target and/or sequences with higher binding affinities compared to a completely random starting library.

The same principle can be broadened to biasing a microarray starting library to contain sequences with increased structural complexity and thus enhanced potential for target binding. In previous works [9–11], starting libraries consisted of 10^2^–10^4^ initial sequences and evolved aptamers through* in silico* genetic algorithms in multiple chip generations (rounds). These studies used either naturally fluorescent targets, utilized a target with well-studied aptamer binding motifs, or took into account the characteristics of known aptamers for library design.

In this work, we applied DNA library patterning in order to circumvent the microarray density problem and rapidly identified an aptamer to the target molecule thrombin in a single round employing a pattern which considered no structural information from previously reported thrombin aptamers. We show that this aptamer does not form the well-known G-quartet structure reported in early iterations of thrombin aptamers by utilizing a combination of imino proton NMR and 2D NMR for structural characterization that is simpler than legacy methods of establishing sequence-specific assignments. The NMR results also raise questions on whether the aptamer identification platform (surface-immobilized or solution-based) may significantly influence the binding mechanism of the final aptamer. These results generally demonstrate a promising method for rapidly identifying and characterizing aptamers, which may directly benefit the field of aptamer biosensors where immobilization of solution-selected aptamers on a sensing platform has proven to be challenging.

## 2. Materials and Methods

### 2.1. Chemicals and Equipment

The chemicals used were IgE (Fitzgerald), Thrombin (Haematologic Technologies, Inc.), BSA and HSA (Sigma), neuropeptide Y (Pheonix Pharmaceuticals), Illustra NAP-25 desalting columns and Cy3 Mono-Reactive Dye Pack (GE Healthcare), NanoDrop (Thermo Scientific), nuclease free water (Gibco). Microarray equipment consisted of the following: custom 8 × 15 k DNA microarrays, 8 × 15 k gasket slides, ozone barrier slides, hybridization chambers, scanner cassettes, hybridization oven, and High-resolution Microarray Scanner (all Agilent) and slide rack and wash dishes (Shandon) and Kimtech polypropylene wipes (Kimberly-Clark). All DNA was purchased through IDT:

 4A018: GGTTGGTTTTTCAATCAGCGATCGCGGAATCCAGGGTTAGGCGGCCAACC (with and without 3′-T_10_-Biotin moiety).
 TFBS: GGTTGGTGTGGTTGG.
 Buffers: Binding [PBSMTB]-1x PBS (8.1 mM Na_2_HPO_4_, 1.1 mM KH_2_PO_4_, 2.7 mM KCl, 137 mM NaCl, pH 7.4) + 1 mM MgCl_2_ + 0.1% Tween-20 and 1% BSA; Washing [PBSM]-1x PBS (8.1 mM Na_2_HPO_4_, 1.1 mM KH_2_PO_4_, 2.7 mM KCl, 137 mM NaCl, pH 7.4) + 1 mM MgCl_2_; Rinse [R]-1/4 dilution of PBSM and nuclease free water.

### 2.2. Protein Labeling

Thrombin was labeled with Cy3 using a Cy3 Mono-Reactive Dye Pack (GE Healthcare). Protein was diluted to 1 mg/mL in 1 mL 0.1 M Na_2_CO_3_ buffer (pH = 9.3) and incubated for 30 minutes. The product was purified with Texas Red protein labeling size exclusion column (Molecular Probes) and the dye-to-protein ratio (D/P) was calculated as 0.8 D/P using the manufacturer's instructions with UV/Vis detection.

### 2.3. Microarray Starting Library Design

UNAFold software was used to screen DNA sequences from the patterned library generated using Perl scripts and to determine which sequences were folding according to a predefined set of criteria. Constraints were set to maximize the number of potential binders: (1) 1st base should be paired with the 50th base; (2) the total number of unpaired bases 10 < unpaired < 30; (3) there should be at least two 4-unpaired base stretches. The secondary structure of generated sequences was evaluated using the UNAFold package with the following set of parameters: *T* = 25°C, [Na^+^] = 100 mM, and [Mg^2+^] = 5 mM. These settings represent generalized conditions related to aptamer studies encompassing a variety of buffers and applications. Only sequences with a secondary structure that passed these selection criteria were candidates for the microarray selection experiments. Sequences were analyzed at random until 50,000 sequences were reported to fit the criteria. Five thousand out of 50,000 total sequences that fit the constraints were randomly incorporated onto the 8 × 15 k microarray chip with a 3′-T_10_ spacer, in duplicate or triplicate. Controls were synthesized with a minimum of 10 replicates also containing a 3′-T_10_ spacer.

### 2.4. Microarray Procedure

Blocking with PBSMTB was performed on the DNA microarray loaded into the gasket for 1 h at room temperature. The slides were quickly edge-tapped to remove excess buffer. Seventy *μ*L Cy3-thrombin (100 nM) in PBSMTB was loaded onto a gasket slide then incubated with the array for 2 hrs at 20°C in a hybridization chamber/hybridization oven. Slides were quickly disassembled in water and washed for 3 min in a PBSM buffer with the slide rack and stir plate then transferred to a 50 mL conical tube with 1/4 PBSM buffer/water for 1 min using a shaker plate. Slides in the slide rack were then dipped in a 50 mL conical tube of nuclease-free water to remove any remaining salt and washed for 1 min on a shaker plate. The microarray slide was slowly withdrawn from the water to promote a drier surface; the back of the slide was wiped with ethanol and then placed in a 50 mL conical tube with a polypropylene wipe at the bottom and centrifuged at 4150 rpm for 3 min. The microarray was loaded into a scanner cassette and covered with an ozone barrier slide before scanning. The arrays were scanned using Agilent Scan Control software. Images (TIFF) were generated using 20-bit imaging at 5 *μ*m (8 × 15 k arrays). Data was extracted using Agilent Feature Extraction software version 10.7.3.1. Mean fluorescence intensity and standard deviation of replicates were determined using code written in Perl. Statistical significance was calculated using a two-tailed *t*-test at 95% confidence interval for potential binders identified through the microarray compared to control sequences. Statistical significance was used as a metric for differentiating real binding events from experimental artifacts.

### 2.5. Surface Plasmon Resonance (SPR)

SPR Biacore studies were carried out on a CM7 (GE Healthcare) chip with neutravidin custom immobilized on the surface. The surface was activated with a mixture of 1-ethyl-3-[3-dimethylaminopropyl]carbodiimide (0.2 M) and N-hydroxysuccinimide (0.05 M) for 420 sec at 10 *μ*L/min. Neutravidin (10 mg/mL; Thermo Scientific) was dissolved in HyClone water then diluted 1/10 in 10 mM sodium acetate buffer (pH = 4.5) and added at 30 *μ*L/min for 210 sec. The surface was blocked with 1 M ethanolamine for 600 sec at 5 *μ*L/min. 3′-biotin-T_10_ 4A018 aptamer was heated to 95°C then introduced at 5 *μ*M in 5 mM MgSO_4_ for 180 sec at 30 *μ*L/min. Samples were diluted to 27.3 *μ*M in HEPES buffer from Biacore (10 mM HEPES, 150 mM NaCl, 0.05% Surfactant P20) and dialyzed into HEPES buffer overnight. The samples were serially diluted to appropriate concentrations then introduced onto the chip at 30 *μ*L/min for 30 sec. Data analysis was performed with BIAevaluation software for three experiments of three replicates for each compound with the background subtracted data (reference channel 1) and plotted in GraphPad Prism 5 using a one site total binding model. Each experiment included freshly prepared reagents introduced onto the same chip in triplicate.

### 2.6. NMR Structural Analysis

Proton NMR of aptamer solutions was performed on a 400 MHz Bruker Avance NMR spectrometer. DNA was dissolved in PBSTMB buffer with H_2_O : D_2_O ratios of 90 : 10 and 0 : 100 at aptamer concentrations of 1 mM for 4A018 and 1.3 mM for the TBA 15 mer. The imino proton spectra were acquired with the W5 water suppression pulse sequence and 2D NOESY spectra were acquired with 1024 points in the direct dimension and 256 points in the indirect dimension with a sweep width of 10 ppm and a mixing time of 0.2 s.

## 3. Results and Discussion

**Figure 1:**
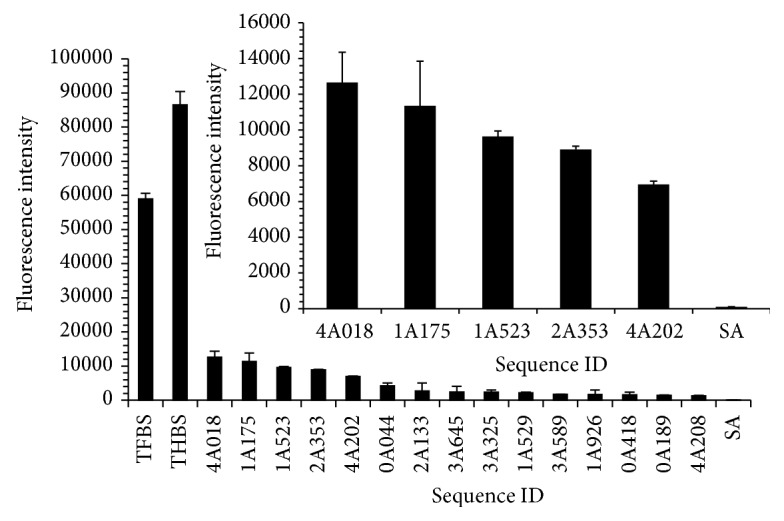
Microarray performance of the top 15 ranked potential thrombin binders of PT2 compared to controls. Inset: close-up view of the intensities of the top 5 ranked sequences compared to the negative control. Error bars represent standard deviation of replicates of fluorescence values obtained from a 2 h incubation of 100 nM Cy3-thrombin with the microarray at 20°C.

A full description of pattern design and analysis is included in Supporting Information text and Figure S1 in Supplementary Material available online at http://dx.doi.org/10.1155/2015/137489. The pattern PT2 was applied to the microarray for assessing thrombin binding. Several potential thrombin aptamer candidates were identified from the microarray work. The top 15 candidates were ranked by fluorescence intensity (Figure 1), in comparison to the positive controls of the reported thrombin fibrinogen binding site aptamer (TFBS) and thrombin heparin binding site aptamer (THBS) [21, 22]. A mutation of the streptavidin aptamer (SA) with an extra guanine base inserted at position 19 was used as a negative control due to its low binding observed with a variety of different targets in preliminary work [23]. All of the top 15 reported sequences demonstrated over 12x higher mean fluorescence intensity values than SA, with the highest intensity sequence, 4A018, reporting mean values over 120x higher than SA. All fluorescence intensity values for the top 15 sequences were statistically significant compared to the SA control (*p* < 0.0001, 95% CI). A close-up of the fluorescence intensity values of the top 5 sequences compared to SA is also shown (Figure 1 inset).

The sequences for each of the top 15 candidates are reported in Table S1, with the predicted secondary structures of the top 5 depicted in Figure S2. The first 6–8 bases of all 15 sequences are similar and also resemble the first 6–8 bases of the TFBS sequence. Previous microarray studies showed a similar trend, with the final thrombin binding sequences after four rounds of mutation also demonstrating a 5′-GGTTGG consensus sequence [9]. While the pattern (PT2) does constrain certain bases in specified positions, it does not account for the abundance of sequence similarity in the first several bases of microarray candidates. Further analysis of the sequences included in the initial library indicated that the number of sequences containing 5′-GGTTGG (3.4% of 5,000 total sequences) was similar to the 3.5% of sequences containing a 5′-GGCCGG, signifying that the library was not biased with an abundance of the motif (see Supporting Information text and Table S2). Random sequences (Table S3) without the 5′-GGTTGG motif did not exhibit significant binding (Figure S3).

To confirm the microarray results due to the low number of technical replicates on the array, the top ranked sequence, 4A018, was assessed for target binding by SPR. Two separate flow channels were immobilized with biotinylated 4A018 with a 3′-T_10_ linker included to permit some flexibility of the sequence and increase the distance of the potential binder from the surface of the SPR chip. Proteins similar in molecular weight and/or also found in the body, bovine serum albumin (BSA), and human serum albumin (HSA), as well as a compound with an isoelectric point (pI) close to that of thrombin, neuropeptide Y (NPY), were assayed for binding in addition to thrombin.

**Figure 2:**
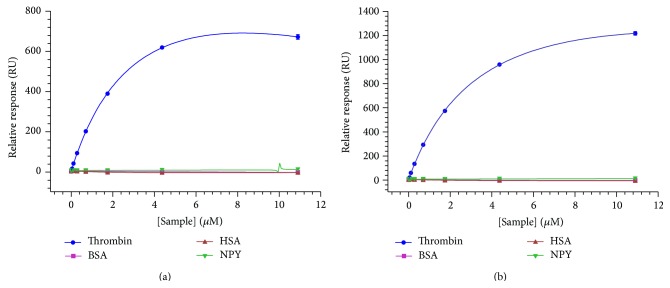
SPR response of 4A018 with analytes in flow channel 3 (a) and flow channel 4 (b). Analytes assayed were thrombin (circle), BSA (square), HSA (triangle), and NPY (downward triangle) at 0–10.9 *μ*M. Error bars represent standard deviation of replicates for the mean of three separate experiments each performed in triplicate.

Thrombin was the only molecule that significantly interacted with 4A018 in either of the flow channels (Figure 2). Flow channel 4 (Figure 2(b)) had an increased relative response compared to channel 3 (Figure 2(a)), likely due to higher immobilization of 4A018 in channel 4 (2482.0 RU) versus channel 3 (1754.6 RU). The curve fit produced dissociation constants (*K*_*d*_) of 4.04 ± 0.31 *μ*M in channel 3 and 3.96 ± 0.29 *μ*M from channel 4 (Figure S4), while BSA, HSA, and NPY demonstrated negligible binding and did not fit the curve (Figures 2 and S4). Flow channels 3 and 4 served as independent replicates confirming the response with each measurement ±1% of the mean *K*_*d*_ (4.00 *μ*M). These SPR results show that 4A018 has affinity for thrombin protein rather than the fluorescent tag used in the microarray experiments and that the interaction is specific to thrombin despite addition of proteins of similar molecular weight and pI. Furthermore, it demonstrates that the use of proper controls and replicates during a microarray experiment enhances the ability to differentiate a real binding event from spurious interactions in a massively parallel format.

One reason the affinity of the microarray selected aptamer may be lower than that of the solution-based aptamers (nanomolar dissociation constants) is because solution-based SELEX is an evolutionary process designed to select the “best” binders by employing multiple rounds of increasingly high stringency conditions. Furthermore, a much smaller starting population was utilized in the microarray experiment, and constraints were imposed that may preclude identification of a sequence with a higher affinity. In addition, the SELEX generated aptamers were fully optimized by truncating primers and nonessential bases and identifying a consensus sequence. Further optimization of patterns and stringency conditions as well as mutational/truncation analysis could lead to aptamers with enhanced target affinity. The reported *K*_*d*_ of the microarray-identified sequence may also be improved under fully optimized binding conditions or by applying genetic algorithms to the lead compound over multiple microarray rounds [9–11]. Refinement of these conditions will be aided by consideration of the sequences of more confirmed thrombin binders from the microarray and by testing more sequences of the patterned libraries following this proof-of-concept work.

In contrast with promoting the sequence as an ideal aptamer, this SPR binding study instead validated the overall hypothesis that a patterned library aids in identifying an aptamer despite the relatively low sequence space covered in a microarray compared to SELEX. The enhanced stringency of the microarray conditions generated a sequence with high target specificity, and a binding sequence was identified by applying the very first pattern designed with no consideration to reported thrombin binders. The binding demonstrated in the SPR experiments shows that an aptamer identified while immobilized on a microarray surface will retain binding when transitioned to a new biosensing platform requiring aptamer surface linkage. Designing a microarray with low replicates of different patterns may serve as a screening mechanism to determine optimal patterns or homologous stretches necessary for target binding. Rather than providing the highest affinity sequence, this proof-of-principle study highlights the use of a computational driven methodology to create a DNA library rather than a SELEX based approach.

Previous reports have shown that the well-known TFBS and THBS thrombin aptamers fold into G-quartet structures [21, 22]. Due to the 5′- and internal (including five GG repeats and one GGG) structural similarity of 4A018 and TFBS, we investigated the possibility that 4A018 also formed a G-quartet structure.

G-quartets have a number of unique features, including in-plane pairing of four guanine bases, slow imino proton exchange, high thermal stability, and* syn* conformations about some guanine glycosidic linkages that can be identified by NMR [24]. While previous studies have used site-specific NMR assignments and multidimensional NMR to determine the three dimensional structure of aptamers [25], we show here that simple screening NMR experiments can be used to rule out G-quartet formation in the 4A018 aptamer.

The imino proton NMR spectra is very sensitive to aptamer folding and the hydrogen bonding patterns resulting from the formation of G-quartets, AT and GC base pairs, loops, and mismatched base pairs [24]. The imino protons exchange rapidly with water and cannot be directly observed unless they are protected by hydrogen bond formation or folded into loops. The lowest energy structure of 4A018 (Figure S2) shows a 7-base pair stem (G1-T7/G44-C50) and a stem starting at C23-G43 with three or more base pairs and three additional base pairs following two non-paired nucleotides. This conformation incorporates two G doublets into stem 1 and two G doublets and a triplet into stem 2, and one of the G doublets is split between stem 1 and stem 2. In addition, each of stems 1 and 2 has two AT base pairs which would be observed in the chemical shift range of 13.5–14.5 ppm rather than the higher field range of 10-11 ppm observed for the T imino protons in short loops. Given these differences, it should be possible to distinguish between G-quartet formation and the calculated structure shown in Figure S2.

**Figure 3:**
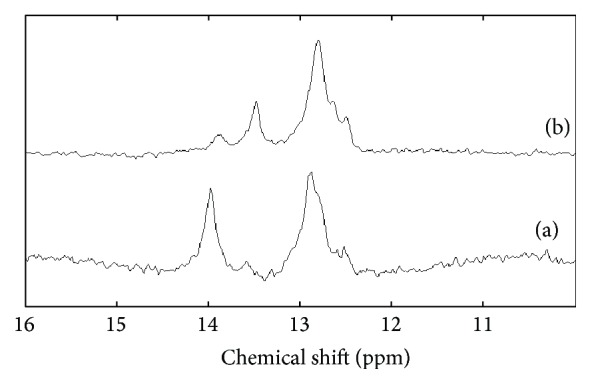
The 400 MHz proton imino spectra of 4A018 at (a) 278 and (b) 298 K.

A comparison of the imino proton spectra of 4A018 at 278 (Figure 3(a)) and 298 K (Figure 3(b)) shows two well resolved peaks that can be assigned to the AT (13.9 ppm) and GC (12.8 ppm) base pairs [26, 27]. The relative AT/GC ratio of the peak areas is ~1 : 1.5 in NMR (Figure 3(a)) which is comparable to the 1 : 1.75 ratio expected from the number of AT and GC pairs in the lowest energy structure (Figure S2). The spectra show significant changes as the temperature increases from 278 to 298 K, which is consistent with the formation of short helical regions as in stem 2, rather than a more stable G-quartet. No peaks are observed in the region between 10 and 12 ppm which contains the signals from bases in protected folds and mismatched base pairs. The two GT mismatches predicted for 4A018 in Figure S2 are located at the end of stem regions and would be difficult to observe by NMR due to solvent exposure. This result suggests that the actual structure is consistent with the predicted structure shown in Figure S2.

Another feature of G-quartets identifiable in the NMR spectra is the* syn* conformation of the glycosidic angle in some of the guanine bases [24]. The* syn* conformation can be detected by 2D Nuclear Overhauser Effect spectroscopy (NOESY) [27] since the cross peak intensities depend on the inverse sixth power of the internuclear distance and the change from the* anti* to the* syn* conformation shortens the distance between the GH8 and GH1′ protons from 3.7 to 2.1 Å. Additionally, software designed to predict oligonucleotide secondary structure is typically unreliable in ability to report the formation of higher order structures (including G-quartets) which can be confirmed by 2D NOESY.

**Figure 4:**
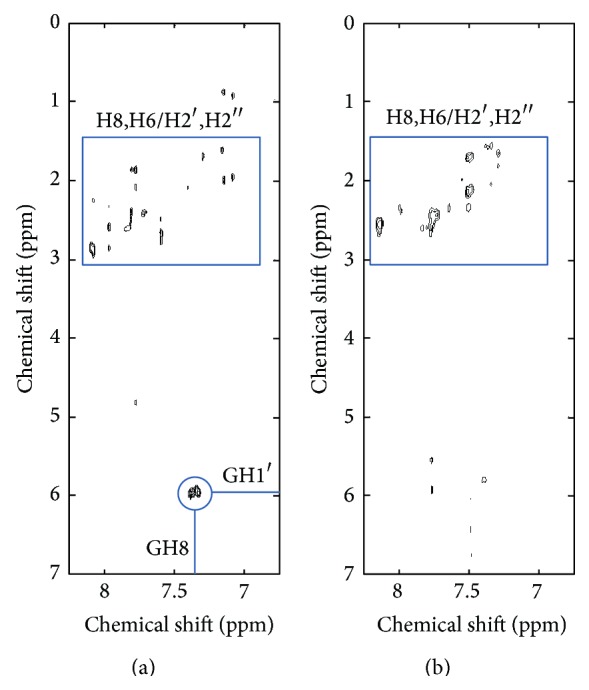
The 2D NOESY NMR spectra for TFBS (a) and 4A018 (b) in D_2_O acquired with a 0.2 s mixing time.

The 2D NOESY spectra for TFBS (Figure 4(a)) and 4A018 aptamer (Figure 4(b)) show the chemical shift correlation between the DNA H8 and H6 base protons (6.5–8.5 ppm) with the H1′ sugar protons (5–6.5 ppm), the H2′ and H2′′ sugar protons (1.8–3 ppm), and the thymine methyl protons (1 ppm) [27]. The G-quartet conformation of the TFBS aptamer has four guanines in the* syn* conformation that give rise to four strong GH8-GH1′ cross peaks enclosed in the circle in Figure 4. The four GH8-GH1′ cross peaks have similar chemical shifts in both dimensions and can be visualized in an expanded view of the spectra (not shown). No strong GH8-GH1′ cross peaks are observed in the 2D NOESY spectra for 4A018, showing that the solution conformation of 4A018 does not contain guanines in the* syn* conformation. This supports the hypothesis that 4A018 does not adopt a G-quartet structure, and it backs the imino proton NMR results suggesting the accuracy of the predicted structure (Figure S2).

The microarray evolution leading to a thrombin aptamer by Platt similarly did not adopt a G-quartet structure [9]. While the microarray work described here and by Platt may essentially preclude this structural feature due to spatial and/or researcher-imposed initial library constraints, it is possible that solution-based methods may promote the selection of G-quartets to thrombin in ways which remain unclear [21, 22, 28]. This aspect of different structures selected depending on the selection mechanism may be of particular interest to researchers intending to apply solution-selected aptamers immobilized on a platform for biosensor design.

## 4. Conclusions

This work illustrates the potential of DNA microarray technology for aptamer identification and highlights patterned libraries designed without prior binding sequence consideration as a viable solution to the limitations on microarray oligonucleotide surface density. This method emphasizes a rational computational driven methodology to DNA library creation rather than a SELEX approach. While the results of these initial proof-of-concept studies may not currently improve upon SELEX in terms of aptamer affinity, the microarrays rapidly provide a starting point to perform additional experiments to generate higher affinity aptamers based on the identified sequences. Binding candidates can be identified and ranked in less than one week utilizing microarray experiments, presenting methodology that is more amenable to potential high throughput applications than traditional SELEX. One area of immediate impact for this methodology is directed at the biosensor field by rendering it feasible to identify a functional aptamer directly immobilized on a solid support to mitigate the variability or elimination of affinity often observed in aptamers that are selected in solution for applications that involve a surface linkage. Furthermore, aptamer based electrochemical or gold nanoparticle biosensing technologies amplify signal and detect analytes at levels orders of magnitude lower than the *K*_*d*_, reducing the reliance of sensor performance on affinity [29, 30]. These platforms also rely on a conformational change of the aptamer to indicate target binding, so knowledge of the structural properties of an aptamer is essential in effective sensor platform design. This work shows that a novel combination of the imino proton NMR and 2D NOESY simplifies screening for aptamer conformation compared to establishing sequence-specific assignments. The NMR studies also demonstrated that the microarray selected aptamer did not form the G-quartet structure common to solution-based SELEX thrombin aptamers. This finding raises consideration that different binding modes may dominate in surface-immobilized aptamer identification strategies in comparison to traditional solution-based SELEX. Therefore, microarray aptamer identification may be* complimentary* to SELEX in the sense that different types of binders could be produced depending on the desired application. Future focus areas include investigating the performance of different patterns including in-depth motif analysis of both binders and nonbinders, as well as the effects of a combined microarray/SELEX scheme.

## Supplementary Material

The Supplementary Material provides additional information on data and methods supporting the Results and Discussion. Initial library pattern design and analysis, sequences and secondary structures of oligonucleotides from Figure 1, analysis of sequence motifs, random oligonucleotide fluorescence intensity measurements and sequences, and surface plasmon resonance sensorgrams are all included in Supplementary Material.

## References

[1] Ellington A. D., Szostak J. W. (1990). In vitro selection of RNA molecules that bind specific ligands. *Nature*.

[2] Tuerk C., Gold L. (1990). Systematic evolution of ligands by exponential enrichment: RNA ligands to bacteriophage T4 DNA polymerase. *Science*.

[3] Stoltenburg R., Reinemann C., Strehlitz B. (2007). SELEX-a (r)evolutionary method to generate high-affinity nucleic acid ligands. *Biomolecular Engineering*.

[4] Jayasena S. D. (1999). Aptamers: an emerging class of molecules that rival antibodies in diagnostics. *Clinical Chemistry*.

[5] Gopinath S. C. B. (2007). Methods developed for SELEX. *Analytical and Bioanalytical Chemistry*.

[6] Berezovski M. V., Musheev M. U., Drabovich A. P., Jitkova J. V., Krylov S. N. (2006). Non-SELEX: selection of aptamers without intermediate amplification of candidate oligonucleotides. *Nature Protocols*.

[7] Savory N., Abe K., Sode K., Ikebukuro K. (2010). Selection of DNA aptamer against prostate specific antigen using a genetic algorithm and application to sensing. *Biosensors and Bioelectronics*.

[8] Cho M., Xiao Y., Nie J. (2010). Quantitative selection of DNA aptamers through microfluidic selection and high-throughput sequencing. *Proceedings of the National Academy of Sciences of the United States of America*.

[9] Platt M., Rowe W., Wedge D. C., Kell D. B., Knowles J., Day P. J. R. (2009). Aptamer evolution for array-based diagnostics. *Analytical Biochemistry*.

[10] Knight C. G., Platt M., Rowe W. (2009). Array-based evolution of DNA aptamers allows modelling of an explicit sequence-fitness landscape. *Nucleic Acids Research*.

[11] Asai R., Nishimura S. I., Aita T., Takahashi K. (2004). *In Vitro* selection of DNA aptamers on chips using a method for generating point mutations. *Analytical Letters*.

[12] Collett J. R., Eun J. C., Ellington A. D. (2005). Production and processing of aptamer microarrays. *Methods*.

[13] Cho E. J., Collett J. R., Szafranska A. E., Ellington A. D. (2006). Optimization of aptamer microarray technology for multiple protein targets. *Analytica Chimica Acta*.

[14] Katilius E., Flores C., Woodbury N. W. (2007). Exploring the sequence space of a DNA aptamer using microarrays. *Nucleic Acids Research*.

[15] Osborne S. E., Ellington A. D. (1997). Nucleic acid selection and the challenge of combinatorial chemistry. *Chemical Reviews*.

[16] Carothers J. M., Oestreich S. C., Davis J. H., Szostak J. W. (2004). Informational complexity and functional activity of RNA structures. *Journal of the American Chemical Society*.

[17] Chushak Y., Stone M. O. (2009). In silico selection of RNA aptamers. *Nucleic Acids Research*.

[18] Davis J. H., Szostak J. W. (2002). Isolation of high-affinity GTP aptamers from partially structured RNA libraries. *Proceedings of the National Academy of Sciences of the United States of America*.

[19] Luo X., Mckeague M., Pitre S. (2010). Computational approaches toward the design of pools for the in vitro selection of complex aptamers. *RNA*.

[20] Ruff K. M., Snyder T. M., Liu D. R. (2010). Enhanced functional potential of nucleic acid aptamer libraries patterned to increase secondary structure. *Journal of the American Chemical Society*.

[21] Bock L. C., Griffin L. C., Latham J. A., Vermaas E. H., Toole J. J. (1992). Selection of single-stranded DNA molecules that bind and inhibit human thrombin. *Nature*.

[22] Tasset D. M., Kubik M. F., Steiner W. (1997). Oligonucleotide inhibitors of human thrombin that bind distinct epitopes. *Journal of Molecular Biology*.

[23] Bittker J. A., Le B. V., Liu D. R. (2002). Nucleic acid evolution and minimization by nonhomologous random recombination. *Nature Biotechnology*.

[24] Macaya R. F., Schultze P., Smith F. W., Roe J. A., Feigon J. (1993). Thrombin-binding DNA aptamer forms a unimolecular quadruplex structure in solution. *Proceedings of the National Academy of Sciences of the United States of America*.

[25] Patel D. J., Suri A. K., Jiang F. (1997). Structure, recognition and adaptive binding in RNA aptamer complexes. *Journal of Molecular Biology*.

[26] Zheng G., Torres A. M., Price W. S. (2008). Solvent suppression using phase-modulated binomial-like sequences and applications to diffusion measurements. *Journal of Magnetic Resonance*.

[27] Wuthrich K. (1986). *NMR of Proteins and Nucleic Acids*.

[28] Kupakuwana G. V., Crill J. E., McPike M. P., Borer P. N. (2011). Acyclic identification of aptamers for human alpha-thrombin using over-represented libraries and deep sequencing. *PLoS ONE*.

[29] Hagen J. A., Kim S. N., Bayraktaroglu B. (2011). Biofunctionalized zinc oxide field effect transistors for selective sensing of riboflavin with current modulation. *Sensors*.

[30] Martin J. A., Chávez J. L., Chushak Y., Chapleau R. R., Hagen J., Kelley-Loughnane N. (2014). Tunable stringency aptamer selection and gold nanoparticle assay for detection of cortisol. *Analytical and Bioanalytical Chemistry*.

