# Supplementary material for: Single-Round Patterned DNA Library Microarray Aptamer Lead Identification
Source: J Anal Methods Chem. 2015 May 14;2015:137489. doi: 10.1155/2015/137489 (PMC4446497; doi:10.1155/2015/137489)
Supplement: Supplementary file 1 — The Supplementary Material provides additional information on data and methods supporting the Results and Discussion. Initial library pattern design and analysis, sequences and secondary structures of oligonucleotides from Figure 1, analysis of sequence motifs, random oligonucleotide fluorescence intensity measurements and sequences, and surface plasmon resonance sensorgrams are all included in Supplementary Material. [file 137489.f1.pdf]

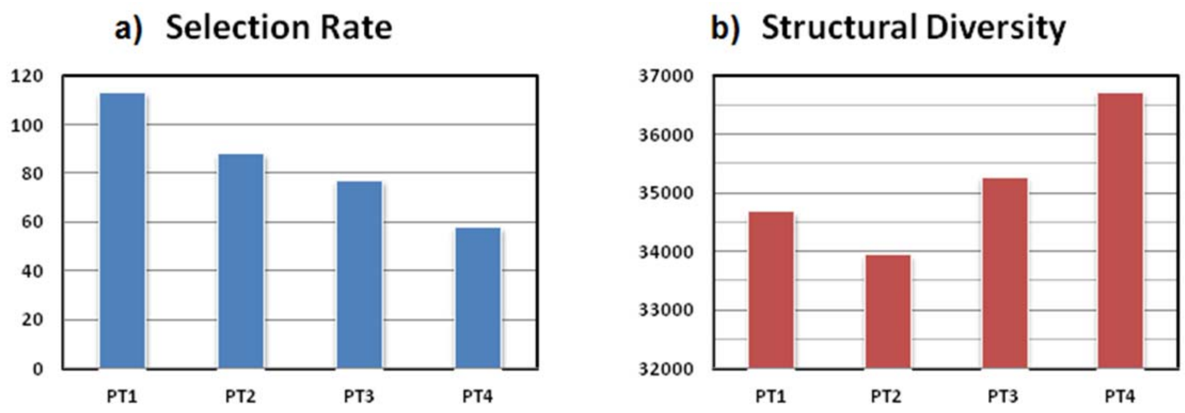

Figure S1. Selection rate (a) and structural diversity (b) of different library patterns.

Library Design. We designed four different patterned libraries of 50nt DNA sequences to test as the initial pool for aptamer selection:

PT1 - $(RY)_3$-N_4-$(RY)_4$-N_3-$(RY)_4$-N_4-$(RY)_4$-N_3-$(RY)_3$

PT2 - $(RRYY)_2$-N_4-(RRYY)-N_3-(RRYY)-N_4-(RRYY)-N_3-(RRYY)-N_4-$(RRYY)_2$

PT3 - $(RRYY)_2$-N_4-(RRRYYY)-N_4-(RRRYYY)-N_4-(RRRYYY)-N_4-$(RRYY)_2$

PT4 - $(RRYY)_2$-N_4-$(RY)_3$-N_4-$(RY)_3$-N_4-$(RY)_3$-N_4-$(RRYY)_2$

R=(A,G), Y=(T,C), N=(A,C,G,T)

The first library (PT1) has a pattern similar to one proposed by Ruff et al [1]. It consists of alternating purines (R=A,G) and pyrimidines (Y=T,C) separated by completely random regions of N_4 and N_3. The second pattern (PT2) was designed to maximize the number of four-way junctions as proposed by Luo et al [2]. Pattern PT1 has 14 completely random bases and $1.8x10^{19}$ possible sequences while pattern PT2 has 18 random bases and $3x10^{20}$ possible sequences. While these patterns are based off of prior designs, the Agilent microarray technology currently only allows for 60 total bases. Employing a T_{10} linker limits the random

region to 50 bases for pattern design. However, in SELEX studies, Ruff et al. used 60 bases and Luo et al., used a 70 base random region, and both included forward and reverse primer regions. [1,2] This discrepancy may result in a different array of structures formed by the microarray patterned libraries compared to the initial design of solution-phase SELEX libraries. Pattern PT3 and PT4 were designed with the same number of random bases, 16, but PT3 allows three of the same purine or pyrimidine in a row as may be observed in complex structures such as G-quartets, while PT4 forces alternation of purines and pyrimidines.

Analysis showed that the different patterns PT1-PT4 have different selection rates and structural diversities (Figure S1). Pattern PT1 has the highest selection rate of 110 (Figure S1a), meaning that only one out of 110 randomly generated sequences with the pattern PT1 satisfies criteria (1) – (3) and can be selected for microarray screening. On the other hand, the pattern PT4 selection rate is almost two times lower, or it requires 58 random sequences to acquire a sequence that passes the selection criteria for microarray screening, and PT4 also has the highest structural diversity. The set of 50,000 sequences generated with the pattern PT4 contains >36,600 different structures, while the set with pattern PT2 has only >33,800 different foldings (Figure S1b). In comparison, for a completely random 50-mer, more than 3000 sequences were screened to select one sequence that satisfies criteria (1) - (3), but the set of 50,000 selected sequences contained more than 45,000 different structural motifs. Therefore, there appears to be a tradeoff between patterned libraries and random libraries, with patterned libraries containing more sequences that will fold into structures with higher complexity, but consisting of lower structural diversity due to the folding constraints imposed by the patterns.

PT2 is the only pattern analyzed in this preliminary work aimed at providing proof-of-principle that a patterned library can address the microarray density problem and identify an

aptamer. Future studies will examine the effects of the different patterns in more detail, and present analysis of pattern design versus target binding activity. The PT2 library was analyzed to determine if it was biased in favor of providing higher percentages of the 5´-GGTTGG sequence than the patterned alternatives. This was accomplished by analyzing the on-chip abundance of several of the possible different fragments within the context of the pattern compared to the TFBS sequence of 5,000 total (Table S2).

Table S1. Sequences of Top 15 Aptamer Candidates and Controls

ID	Sequence
4A018	GGTTGGTTTTTCAATCAGCGATCGCGGAATCCAGGGTTAGGCGGCCAACC
1A175	GGTTGGTTTATTGGTTTATGGCCGCGCAGCCATCAGTCCTAGGGCCGACC
1A523	GGTTGGCTCGGGAGCTCCTGACCCGTGGGTTGGAAATTAAAAAGCCGGCC
2A353	GGTTGGTCATTAAATTGGAAATTGCGGAGCTGACAATTAGAAAGCCGGCC
4A202	GGCTGGTCGGTTGGTTAGTAGTCTCGCGGCCGCGGGTCCCATAACCAGCC
0A044	GGTTGGTTTGACGGTTTGTGGTCCGATGATTAGAGATCACAAAACCGACC
2A133	GGTTGGTCTACAAGCCGGTGACCTGGCGGCTGCTGATTCGCAAGTCAACC
3A645	GGCTGGCTTTGCGGTTGGTAATCCTGAGACTGAAGATCAGGCGATCAGCC
3A325	GGTTGGTCTTTGAGCCTCTGGTCAGGAGGTCTTCGACTCATGAGCCGACC
1A529	GGTTGGTTCGGGGGCTGAGGGTCGCCGAATTGATAGTTAGTTGGCCGACC
3A589	GGTTGGTCTCTAAATCATCAGCCAATAGATCTGAAGCCTCCTGACCGGCC
1A926	GGTTGGTCGCTGAACTTCGGATTGTAAGGCTCGGAACTTACTGGTCAACC
0A418	GGTTGGTTGAATAATTTCCAGTCAGGAGACTGAAAATCGCATGGCCAGCC
0A189	GGTTGGCTTTCTAATCACTGGTTTAGTGATTCCAAATTCTCTGATCAACC
4A208	GGTTGGCTCCTAAATCTATAACTCGTAAATCCGCAATTCGAGGGCTAACC
TFBS	GGTTGGTGTGGTTGG
THBS	AGTCCGTGGTAGGGCAGGTTGGGGTGACT
SA	TCTGTGAGACGACGCACCGGTCGCAGGTTTTGTCTCACAG

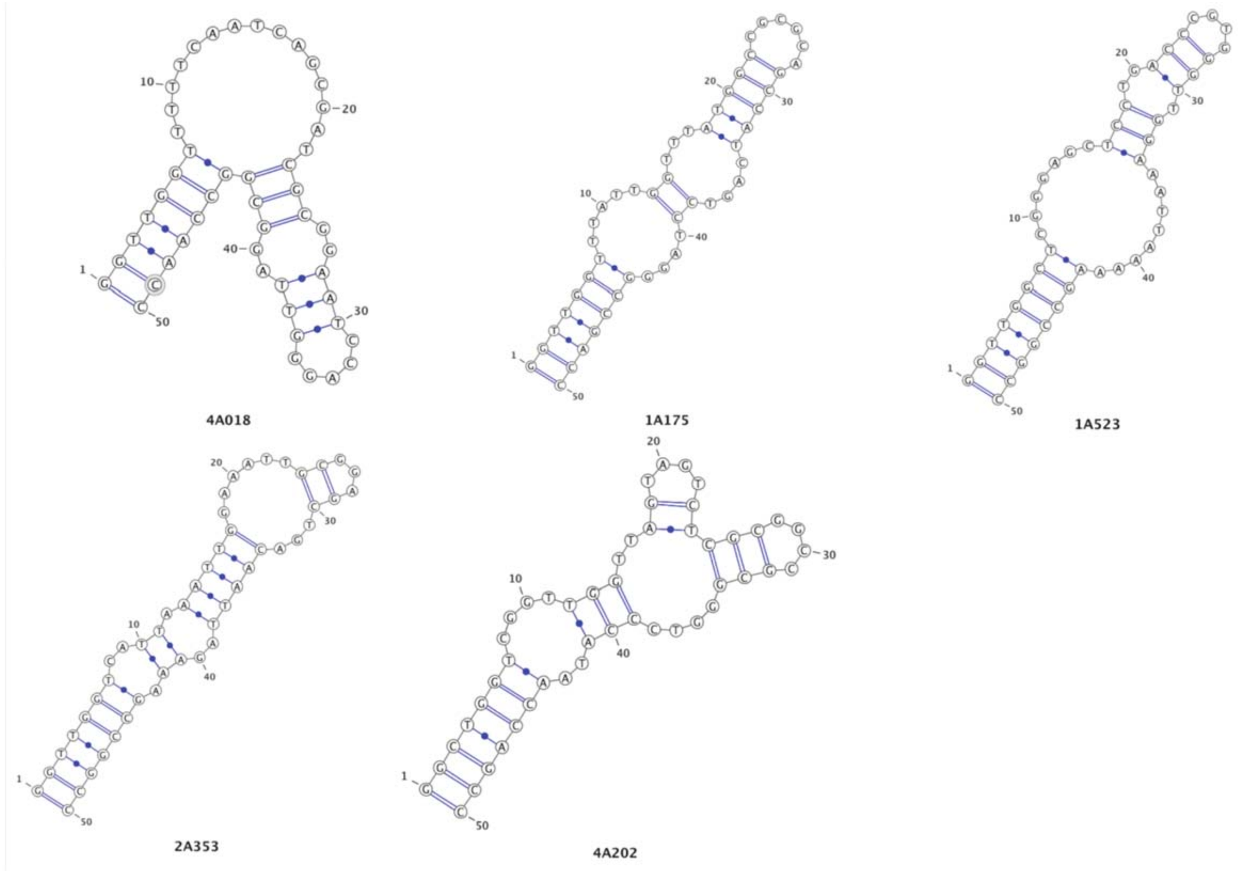

Figure S2. Secondary structures of top 5 ranked sequences from microarray. The structure of the core aptamer region did not change when the T_{10} linker was included in analysis.

Table S2. PT2 Library Analysis

5´ Fragment	Counts	% Total
GGTTGG	170	3.4
GGTTGGT	95	1.9
GGTTGGTT	44	0.9
GGCCGG	176	3.5
GGCCGGC	90	1.8
GGCCGGCC	38	0.8
GGTGTGG	0	0.0
TFBS: GGTTGGTGTGGTTGG		

The 6 base GGTTGG present in the TFBS accounts for 170 of the PT2 sequences, or 3.4% of the 5,000 total oligonucleotides. Expanding this to the first 7 bases of TFBS (GGTTGGT) means that 1.9% (95) of the library present contained this 5´ sequence. When compared to the sequences with C's substituted for T's (positions 3-4 and 7-8), GGCCGG and

GGCCGGC constitute 3.5% (176) and 1.8% (90), respectively. Therefore, the sequences which begin with GGTTGG as in the TFBS do not dominate the patterned library, and the fact that the top 15 ranked sequences all contain this motif (with the exception of a C in position 3 for 4A202 and 3A645) is an indicator of the importance of this sequence to thrombin binding. Sequences with the GGTTGGTG or GGTGTGG motif were not present because the PT2 pattern does not allow for G at positions 4 or 8. Homology is also observed in the final 6 bases of all of the sequences, as well as internally with A stretches appearing as a common motif in several of the sequences (Table S1).

Analysis of the sequences of oligonucleotides with sequence ID numbers appearing in numerically consecutive order directly following the top 5 sequences (Table S3) shows that the sequences of all bases were random, and certain "blocks" were not designated to consist of the potential binding GGTTGG variant. Sequence 1A176 exhibited a single higher intensity reading causing high standard deviation of the sequence. However, none of the random sequences demonstrated significant binding compared to 4A018 (Figure S3 inset v. Figure 1 inset).

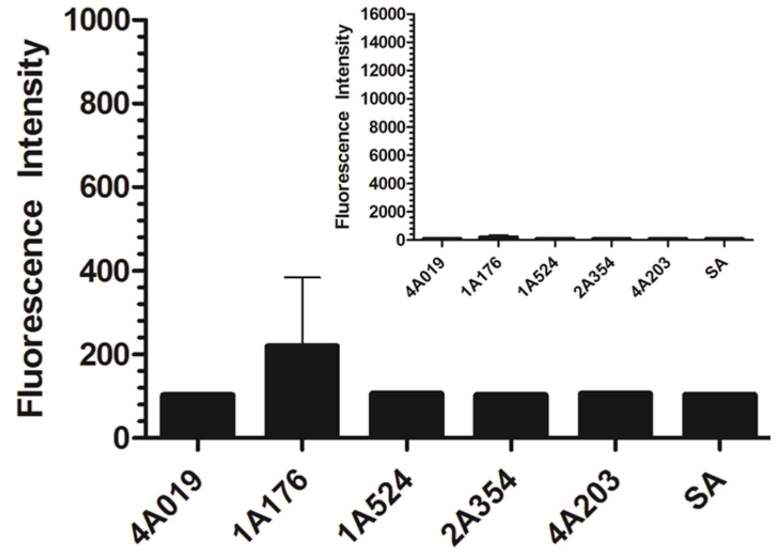

Figure S3. Random sequences from PT2 compared to SA negative control. Error bars represent standard deviation of replicates. Figure inset is scaled to that of the top binding sequences depicted in Figure 1 inset for comparison.

Table S3. Sequences of Random Oligonucleotides from PT2

ID	Sequence
4A019	AACCAGTTATCGAGTCGGCAGTCTGCTGATCTAAGACCACCTGGCTGGTT
4A203	AACCGATTAGCAAGCTTATGATCTATAAGTCTGCGATTGAACAATCGGTT
2A354	GGCCGACCTGGAGGCTATAGGCCCTCAAGCCTCCAGTCTAAGGATTGGCC
1A176	GGTTAATCCCGTGGCTTTTAACCGTGTGGTCCTGGGCCCCGCAGTTAACC
1A524	GACTGGTTCCATAGCTCTCAGCTACACGGTTACTAGTTTGGCGGCCAGTC

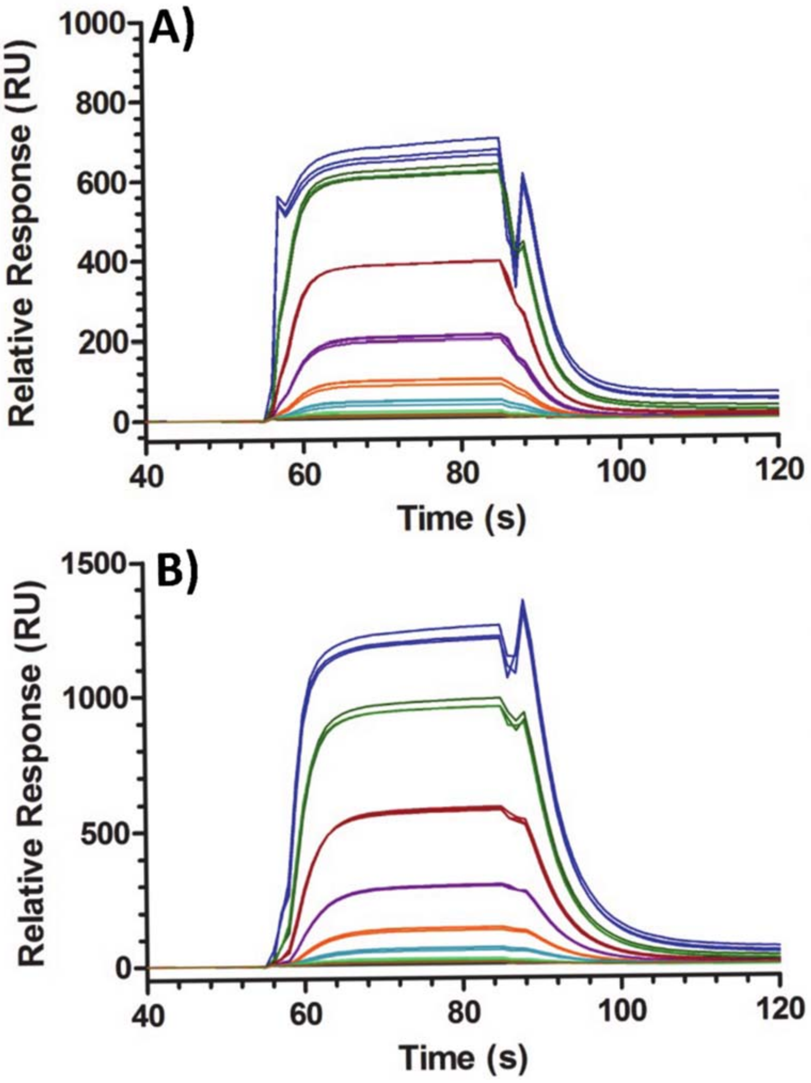

Figure S4. Background subtracted and baseline normalized sensorgrams of a serial dilution of thrombin for (A) channel 3 and (B) channel 4. The average is plotted for each of three

experiments performed in triplicate. Data was evaluated using BIAevaluation software and plotted in GraphPad Prism 5.

3´-biotin-T_{10} linkers were used to immobilize 4A018 to the SPR surface. The T_{10} linker was included in order to increase the distance of the aptamer from the SPR surface and increase flexibility, which has been reported to enhance the affinity relative to the native sequence [3-4]. The linker also mimics the original aptamer identification conditions on the microarray in order to more accurately reproduce relevant experimental conditions.

Notes and references

[1] K. M. Ruff, T. M. Snyder, and D. R. Liu, “Enhanced functional potential of nucleic acid aptamer libraries patterned to increase secondary structure,” *J. Am. Chem. Soc.*, vol 132, pp. 9453– 9464, Jul 14 2014.

[2] X. Luo, M. McKeague, S. Pitre, M. Dumontier, J. Green, A. Golshani, M. C. Derosa, and F. Dehne, “Computational approaches toward the design of pools for the in vitro selection of complex aptamers,” *RNA*, vol 16, pp. 2252-2262, November 2010.

[3] C. Polonschii, S. David, S. Tombelli, M. Mascini, AM. Gheorghiu, “A novel low-cost and easy to develop functionalization platform. Case study: Aptamer-based detection of thrombin by surface plasmon resonance,” *Talanta*, vol 80, pp. 2157-2164, 2010.

[4] Q. Tang, X. Su, K. P. Loh, “Surface plasmon resonance spectroscopy study of interfacial binding of thrombin to antithrombin DNA aptamers,” *J. Colloid and Interface Science*, vol 315, pp 99-106, 2007.
